# miR-200c inhibits breast cancer proliferation by targeting KRAS

**DOI:** 10.18632/oncotarget.5198

**Published:** 2015-09-09

**Authors:** Cailu Song, Long-Zhong Liu, Xiao-Qing Pei, Xiaoping Liu, Lu Yang, Feng Ye, Xinhua Xie, Jianping Chen, Hailin Tang, Xiaoming Xie

**Affiliations:** ^1^ Department of Breast Oncology, Sun Yat-Sen University Cancer Center, State Key Laboratory of Oncology in South China, Collaborative Innovation Center for Cancer Medicine, Guangzhou, Guangdong, China; ^2^ Department of Ultrasond, Sun Yat-Sen University Cancer Center, Guangzhou, Guangdong, China; ^3^ School of Chinese Medicine, The University of Hong Kong, Hong Kong

**Keywords:** microRNA, miR-200c, KRAS, breast cancer, proliferation

## Abstract

The microRNA, miR-200c, is involved in the tumorigenesis and progression of a variety of cancers. The purpose of this study was to investigate the expression, mechanism and prognostic roles of miR-200c in breast cancer. We found that miR-200c was downregulated in both breast cancer tissue and cell lines using quantitative real-time PCR (qRT-PCR). *In situ* hybridization (ISH) and microarrays showed that low miR-200c expression was associated with poor patient overall survival (OS) and disease free survival (DFS). We used luciferase reporter plasmids to find that miR-200c inhibited the AKT and ERK pathways by directly targeting KRAS. Repression of KRAS by miR-200c suppressed the proliferation and survival of breast cancer cells *in vitro* and *in vivo*. miR-200c also had an anti-tumor effect by negatively regulating KRAS in a xenograft mouse model. Our findings provide clues regarding the role of miR-200c as a tumor suppressor in breast cancer through the inhibition of KRAS translation both *in vitro* and *in vivo*. miR-200c could be a potential therapeutic target in breast cancer.

## INTRODUCTION

Breast cancer is the most common cancer and holds the highest mortality in females worldwide [1]. Breast cancer is a heterogeneous disease. According to its molecular subtype, breast cancer can be classified into different categorizations that provide an efficient path for comprehensive therapy and prognosis [2]. Specifically, these types are luminal A type (ER+ and/or PR+, HER2-), luminal B type (ER+ and/or PR+, HER2+), HER2 over-expressing type (ER−, PR−, and HER2+) and basal-like type (ER-, PR-, HER2-, cytokeratin 5/6+, and/or EGFR+) [3]. Although the detection and treatment of breast cancer has greatly improved, prognosis for most patients is still poor [4].

MicroRNAs (miRNAs) are small single-stranded RNAs that have various vital biological functions [5]. They are highly conserved and specific, regulating gene expression by binding to the 3′-untranslated region (3′-UTR) of target messengerRNAs (mRNAs) and either inhibiting translation or inducing degradation of mRNAs [6]. The microRNA-200 (miR-200) family consists of miR-200a, miR-200b, miR-200c, miR-141 and miR-429, which all have the same seed sequence and homologous targets. The miR-200 family can regulate the epithelial-to-mesenchymal transition (EMT) and enhance the reverse of this process [7]. The dysregulation of the miR-200 family is involved in several malignancies, such as gastric [8, 9], breast [10], bladder [11], and lung [12] cancer. Among all the miR-200 family members, miR-200c is considered to play an important role in EMT [13] and cancer chemo-sensitivity [14, 15]. By regulating a multitude of oncogenic pathways, miR-200c is a potent inhibitor of tumor progression and therapy resistance [16]. However, the prognostic role of miR-200c in breast cancer is not completely known.

RAS is a small gene family known for its ability to cause neoplasia. There are three *ras* genes that have been identified in the mammalian genome, designated HRAS, KRAS and NRAS [17]. RAS can be activated by retroviral insertional mutagenesis which has been described in the origin of avian [18] and mammalian [19] tumors. The genetic mutation of intracellular effectors that are involved in GTPase-related signaling pathways usually have an effect on the response of the targeted therapy [20]. Recently, KRAS mutations have been reported to be involved in human cancer, including nasopharyngeal carcinoma [21], ovarian cancer [22], lung cancer [23], pancreatic cancer [24], oral squamous cell carcinoma [25], esophageal squamous cell carcinoma [26], colorectal cancer [27], breast cancer [28] and so on.

Although abnormal expression of miRNA and KRAS has been associated with tumorigenesis in human breast cancer, we still know little about how miRNAs act on KRAS. In this study, we investigated the expression of miR-200c and its potential prognostic role in breast cancer patients. We predicted and verified KRAS as a target of miR-200c in breast cancer. Furthermore, we confirmed that miR-200c could inhibit KRAS expression and suppress the proliferation and clone formation of breast cancer cells *in vitro*. miR-200c over-expression could inhibit the tumor formation and growth *in vivo*.

## RESULTS

### miR-200c is downregulated in breast cancer cell lines and clinical specimens

**Figure 1:**
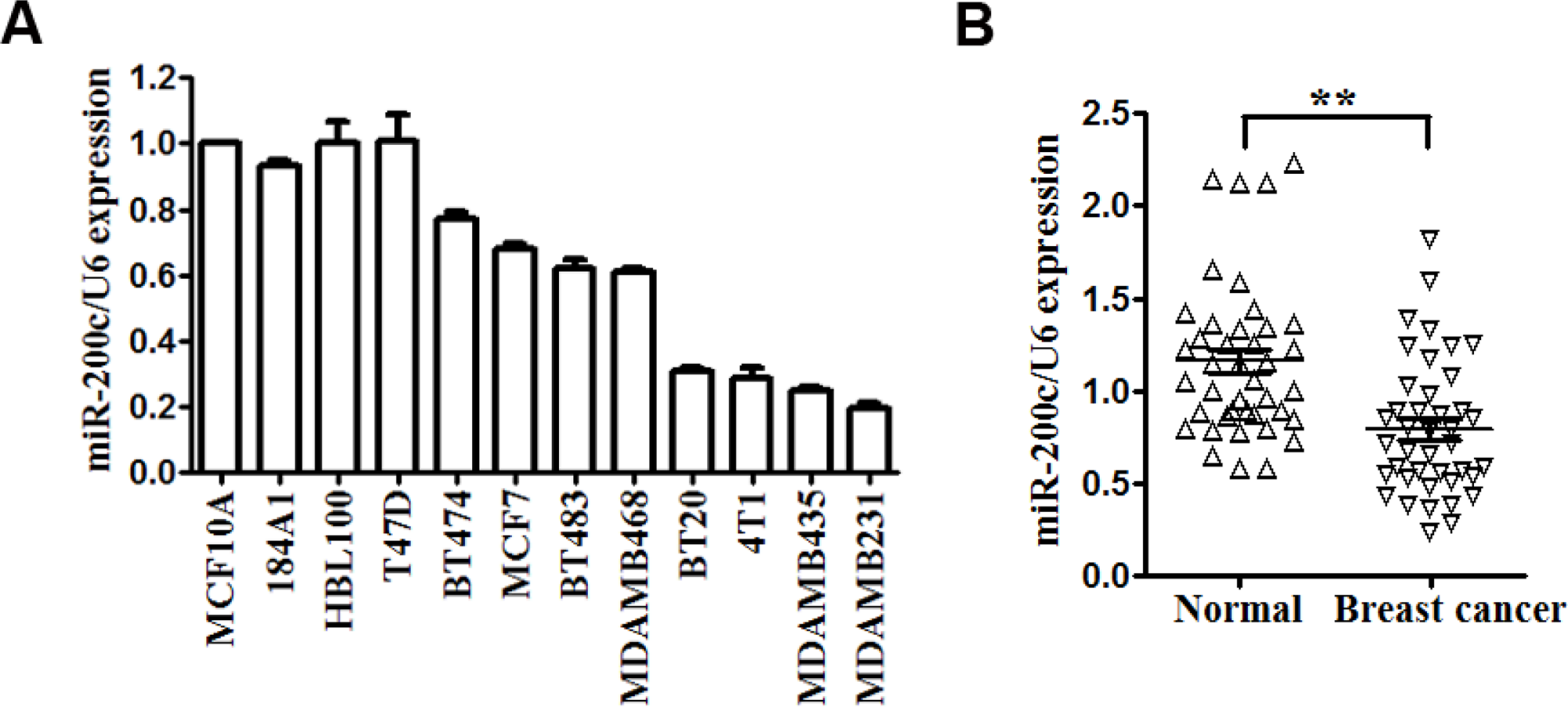
miR-200c is downregulated in breast cancer cell lines and clinical specimens **A.** Expression levels of miR-200c determined by qRT-PCR in 12 different mammary cell lines, including 3 human mammary epithelial cell lines, 8 human breast cancer cell lines and 1 mouse breast cancer cell line. miR-200c expression was normalized using U6 RNA expression. **B.** Expression levels of miR-200c in 41 pairs of breast cancer tissues (BC) and their matched normal adjacent tissues (CTR). All of the data are shown as the means ± s.e.m. **P < 0.01.

miR-200c is reported to be downregulated in many cancers, including gastric cancer [29], renal cell carcinoma [30], ovarian cancer [31] and so on. qRT-PCR analysis was used to detect the expression of miR-200c in 12 different mammary cell lines, including human mammary epithelial (HME) cell lines (MCF-10A, 184A1, BHL-100), human breast cancer cell lines (T47D, BT-474, MCF-7, BT-483, MDA-MB-468, BT-20, MDA-MB-435, MDA-MB-231) and mouse breast cancer cell line 4T1. Compared with HME cell lines, we found that miR-200c was downregulated in human and mouse breast cancer cell lines, especially in BT-20, MDA-MB-435, MDA-MB-231 and 4T1 (Figure 1A). We chose MDA-MB-231 and 4T1 for further studies.

Then, we detected the expression of miR-200c and KRAS in 41 pairs of breast cancer tissues (BC) and their matched normal adjacent tissues (CTR). Among 41 breast cancer patients, approximately 80% (*P* < 0.0001, 33 of 41 patients) of tumor samples revealed reductions in miR-200c levels (Figure 1B). As show in Supplement Figure S1, 87.8% (*P* < 0.0001, 36 of 41 patients) of tumor samples revealed an increase in the expression levels of KRAS. Furthermore, statistical analysis revealed that miR-200c levels in 41 breast cancer tumors samples were significantly inversely correlated with the expression of KRAS (*r* = −0.64, *P* < 0.001; Supplement Figure S2). These results indicate that downregulation of miR-200c is a frequent event in breast cancer clinical specimens and could be related to the carcinogenesis of breast cancer.

### Decreased miR-200c levels are correlated with clinical stage, relapse, metastasis and poor clinical outcomes

We explored the potential clinicopathological implications arising from altered miR-200c levels. The tissue microarrays (TMAs) of 134 breast cancer samples were used for *in situ* hybridization analysis. These clinical samples were divided into low or high expression groups based on miR-200c expression scores less or greater than 2 (Table 1). We found that miR-200c levels were inversely correlated with clinical stage, local relapse and distant metastasis (*P* = 0.017, 0.019 and 0.033 respectively) (Table 1) but not correlated with age, menopause, tumor size, lymph node metastasis, ER, PR, HER2 and P53 status among the 134 breast cancer patients. These results revealed that miR-200c might play a vital role in the occurrence and progression of breast cancer.

**Table 1:** Clinicopathological variables and miR-200c expression in 134 breast cancer patients

Characteristics	Total (*n* = 134)	miR-200c low (*n* = 83)		miR-200c high (*n* = 51)		*P* value
		No.	%	No.	%	
OS						0.001*
Present	107	59	55.1	48	44.9	
Absent	27	24	88.9	3	11.1	
DFS						0.002*
Present	96	53	55.2	43	44.8	
Absent	38	30	78.9	8	22.1	
Age (years)						0.397
<50	82	52	63.4	30	36.6	
> =50	52	31	59.6	21	42.3	
Menopause						0.294
Yes	63	37	58.7	26	41.3	
No	71	46	64.8	25	35.2	
Tumor size (cm)						0.491
= <2	38	23	60.5	15	39.5	
>2	96	60	62.5	36	37.5	
LNMET						0.076
Yes	80	54	67.5	26	32.5	
No	54	29	53.7	25	46.3	
TNM stage						0.017*
I–II	78	42	53.8	36	46.2	
II–IV	56	41	73.2	15	26.8	
Local relapse						0.019*
Yes	8	8	100.0	0	0	
No	126	75	59.5	51	40.5	
Distant metastasis						0.033*
Yes	34	26	76.5	8	23.5	
No	100	57	57.0	43	43.0	
ER status						0.222
Positive	51	29	56.9	22	43.1	
Negative	83	54	65.1	29	34.9	
PR status						0.099
Positive	55	30	54.5	25	45.5	
Negative	79	53	67.1	26	32.9	
HER-2 status						0.529
Positive	22	14	63.6	8	36.4	
Negative	112	69	61.6	43	38.4	
P53 status						0.156
Positive	77	51	66.2	26	33.8	
Negative	57	32	56.1	25	43.9	

* means statistically significant (*P* < 0.05).

% means percentage within the row.

**Figure 2:**
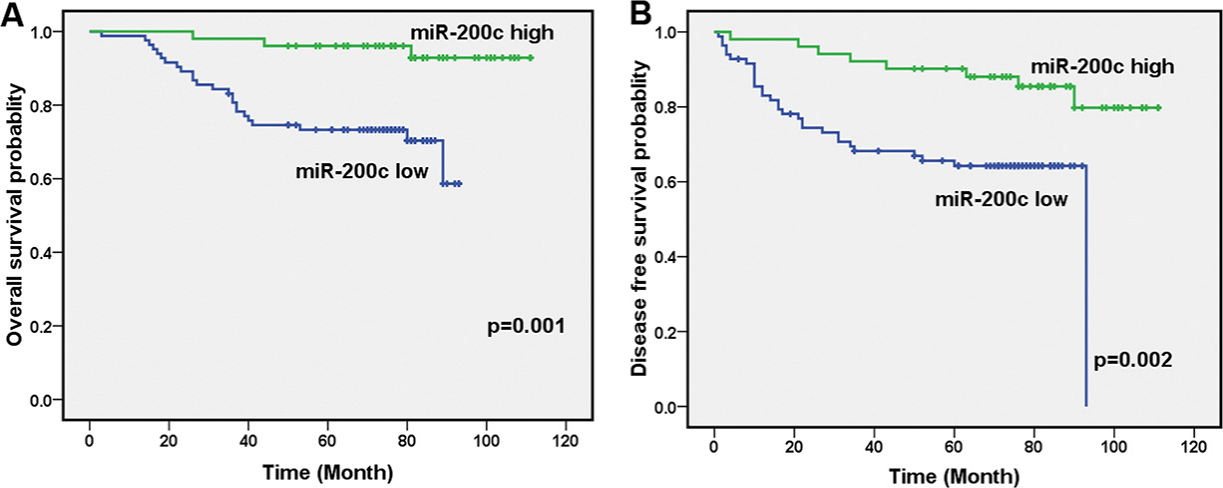
Decreased miR-200c levels are correlated with clinical stage, local relapse, distant metastasis and poor clinical outcomes **A.** Low levels of miR-200c correlated with shorter survival. OS curves for 134 studied patients with high or low miR-200c expression. **B.** DFS curves for 134 studied patients with high or low miR-200c expression.

To explore the significance of miR-200c in clinical prognosis, we used Kaplan-Meier survival analysis to make patient overall survival (OS) and disease free survival (DFS) curves. The results demonstrated that patients with low miR-200c expression had shorter mean months of OS and DFS than patients with high expression (*P* = 0.001 for OS, *P* = 0.002 for DFS, Figure 2A and 2B). These results demonstrate that miR-200c expression is significantly associated with patient OS and DFS.

### miR-200c inhibits the AKT and ERK pathways by targeting KRAS

**Figure 3:**
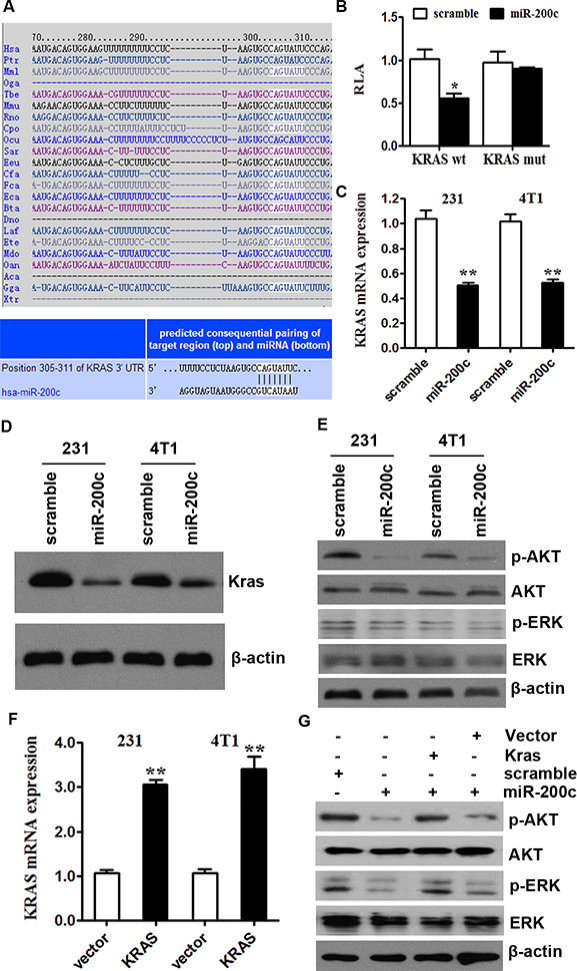
miR-200c inhibits the AKT and ERK pathways by targeting KRAS **A.** miR-200c bound to the 3′-UTRs of KRAS. Predicted binding between miR-200c and the seeds matched in the 3′-UTRs of predicted genes. **B.** Luciferase assay of MDA-MB-231 and 4T1 cells cotransfected with miR-200c mimics, a scrambled control and a luciferase reporter containing KRAS 3′-UTR (KRAS-wt) or mutant constructs in which the first four nucleotides of the miR-200c binding site were mutated (KRAS-mut). All of the data are shown as the means ± s.e.m. *P < 0.05. **C.** MDA-MB-231 and 4T1 cells were transfected with miR-200c or scramble in control. miR-200c overexpression inhibited the KRAS mRNA expression. All of the data are shown as the means ± s.e.m. ** *P* < 0.01. **D.** MDA-MB-231 and 4T1 cells were transfected with miR-200c or scramble in control. miR-200c overexpression inhibited the protein expression of KRAS. **E.** MDA-MB-231 and 4T1 cells were transfected with miR-200c or scramble in control. miR-200c overexpression inhibited the activity of AKT and ERK pathway. **F.** The transfection of KRAS was successful. All of the data are shown as the means ± s.e.m. ** *P* < 0.01. **G.** miR-200c/scramble control transfection followed by KRAS/mock vector transfection 24 hours later in 4T1 cells affected AKT and ERK signalling. The protein expression levels of phosphorylated AKT (pAKT) and ERK (pERK) were measured to examine the activity of AKT and ERK pathways. The miR-200c-induced downregulation of KRAS was rescued by the introduction of KRAS. A similar alteration was observed in the phosphorylation levels of AKT and ERK.

To explore the function of miR-200c, we used computational algorithms (Targetscan and Miranda) to identify the potential target gene of miR-200c in human breast cancer. The algorithm predicted that KRAS was a target gene of miR-200c (Figure 3A). We performed luciferase reporter assays to clarify this finding. The full-length KRAS was cloned downstream of the firefly luciferase gene and cotransfected with miR-200c mimics or scrambled oligonucleotides controls (The transfection of miR-200c or scramble was successful, Supplement Figure S3). Luciferase activity was measured 48 hours after transfection. We found that the luciferase expression was decreased in MDA-MB-231 cells that were co-transfected with miR-200c and the wild-type KRAS 3′-UTR, compared to controls. However, no decrease can be observed in cells which were co-transfected with miR-200c and the mutant KRAS 3′-UTR in comparison with controls (Figure 3B). These results suggest that miR-200c can directly target KRAS.

To determine the level at which miR-200c influences KRAS expression, we examined the expression of KRAS mRNA after transfection with miR-200c mimics or scramble doligonucleotides controls in MDA-MB-231 and 4T1 cells. After transfection with miR-200c mimics, the expression of KRAS mRNA in two cell lines was lower than in the controls (Figure 3C). The effect of miR-200c on KRAS expression was also verified by Western blotting analyses. The over-expression of miR-200c reduced KRAS protein levels significantly (Figure 3D). These results provide evidence that miR-200c directly recognizes the 3′-UTR of KRAS mRNA and inhibits KRAS translation.

The activation of KRAS can trigger several significant pathways, including the AKT and ERK pathways [32, 33]. Therefore, we presumed that miR-200c might regulate those pathways by targeting KRAS. Then we upregulated miR-200c levels via miR-200c mimics in MDA-MB-231 and 4T1 cells. Western blot results demonstrate that miR-200c decreased the phosphorylation levels of AKT and ERK and negatively regulated the pathways (Figure 3E, quantification of protein levels are supplied in Supplement Figure S4).

Subsequently, we treated 4T1 cells with miR-200c or scramble control. 24 hours later we transfected them with KRAS-encoding vector (without an endogenous 3′-UTR) or mock vector (Figure 3F showed that the transfection was successful in both MDA-MB-231 and 4T1 cells). The miR-200c mimic induced downregulation of KRAS, then the downregulation was rescued by the introduction of KRAS. A similar alteration was observed in the phosphorylation levels of AKT and ERK. The downregulation of phosphorylated AKT and ERK by miR-200c could also be rescued by re-expression of KRAS in 4T1 cells (Figure 3G; quantification of protein levels are supplied in Supplement Figure S5). These results suggest that miR-200c inhibits the AKT and ERK pathways by targeting KRAS.

### miR-200c inhibits the proliferation and colony formation of breast cancer by targeting KRAS

To analyze the biological consequences of the miR-200c-driven repression of KRAS in breast cancer cells, we investigated whether increasing or decreasing the expression of miR-200c would have an impact on cell proliferation and clone formation in MBA-MD-231 and 4T1 cells.

**Figure 4:**
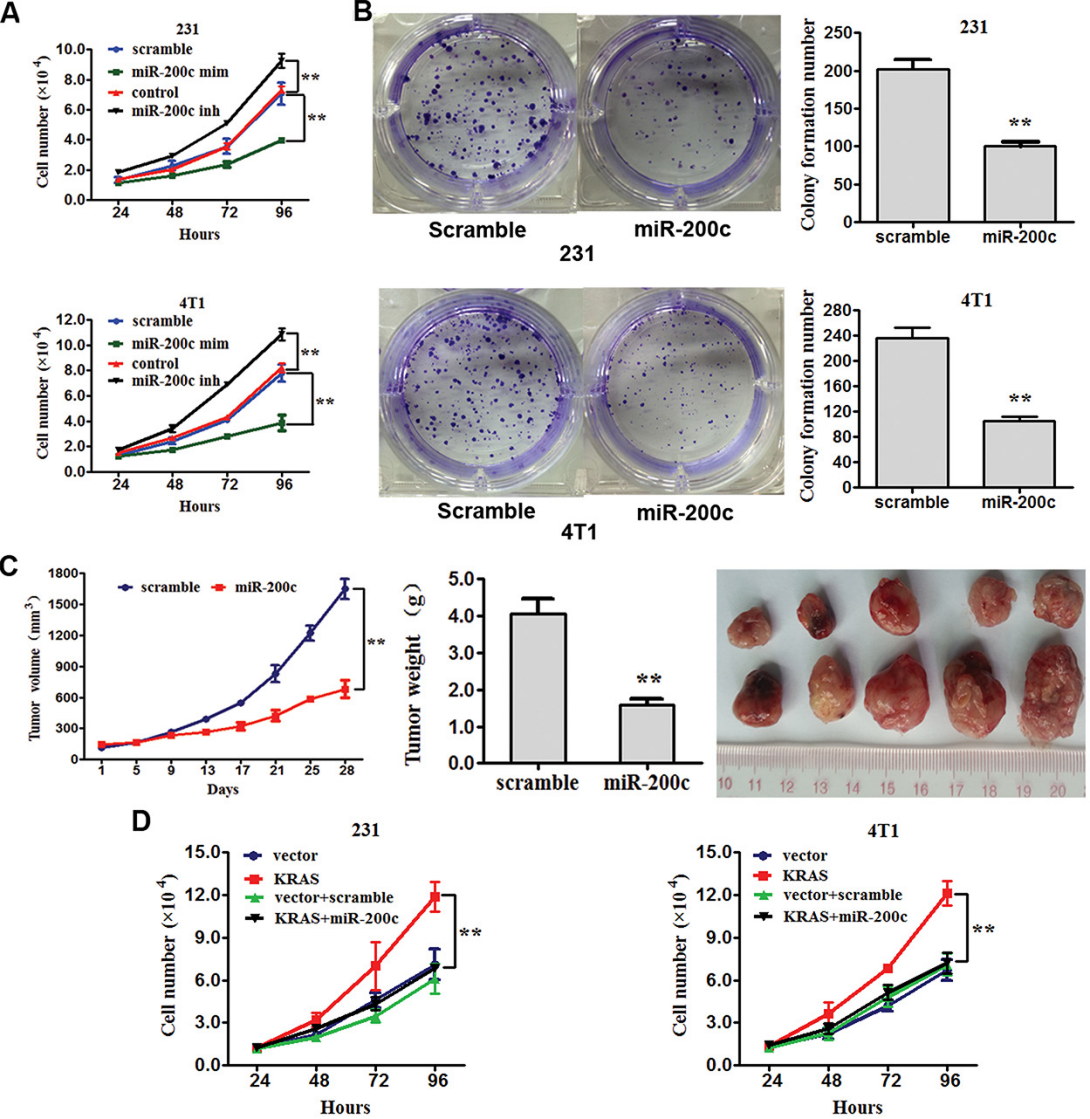
miR-200c inhibits the proliferation and clone formation of breast cancer by targeting KRAS **A.** MDA-MB-231 and 4T1 cells were transfected withmiR-200c mimics, miR-200c inhibitors or their scrambled oligonucleotides controls. The proliferation of cells was assayed. All of the data are shown as the means ± s.e.m. **P < 0.01. **B.** MDA-MB-231 and 4T1 cells were transfected with scramble or miR-200c. Clone formation of cells was examined. Numbers of colonies are showed as the means ± s.e.m. **P < 0.01. **C.** Tumor growth in mouse xenograft models. 4T1 cells transfected with recombinant lentiviruses containing miR-200c precursor or scramble sequences were injected subcutaneously into BALC/c mice (five in each group). The tumor volume was measured every four days. After 28 days, the mice were killed, necropsies were performed, and the tumors were weighed. All of the data are shown as the means ± s.e.m. **P < 0.01. **D.** MDA-MB-231 and 4T1 cells were transfected with mock vector, scrambled oligonucleotides controls, KRAS-encoding vector + miR-200c or mock vector + scrambled oligonucleotides controls respectively. The proliferation of cells was assayed. All of the data are shown as the means ± s.e.m. **P < 0.01.

MDA-MB-231 and 4T1 cells were transfected with miR-200c mimics, miR-200c inhibitors or their scrambled oligonucleotides controls respectively (The transfection were successful, see in Supplement Figure S3) and counted after 24 hours. The proliferation assay showed that the increased expression of miR-200c in MDA-MB-231 and 4T1 cells attenuated proliferation compared with the control cells, while the decreased expression of miR-200c promoted proliferation (Figure 4A). Moreover, increased expression of miR-200c inhibited the ability to form clones in soft agar (Figure 4B). We then decided to evaluate the effect of miR-200c over-expression on tumor formation and growth *in vivo* by adopting a xenograft model of breast cancer cells in BALC/c mice. 4T1 cells infected with recombinant lentiviruses containing miR-200c precursor or scrambled sequences were subcutaneously injected into flank BALC/cmice (five in each group). The tumor volume was measured every four days after injection and accordingly the growth curves of the tumors were plotted. All mice were later sacrificed to harvest the xenograft. The mean volume and weight of tumors was reduced in the miR-200c overexpressing group compared with the control group (Figure 4C).

The above results prompted us to validate that miR-200c could indeed inhibit the proliferation of breast cancer by repressing KRAS expression. For this purpose, MDA-MB-231 and 4T1 cells were transfected with mock vector, scrambled oligonucleotides controls, KRAS-encoding vector + miR-200c or mock vector + scrambled oligonucleotides controls respectively. The results show that increased expression of miR-200c inhibited proliferation by repressing KRAS (Figure 4D).

## DISCUSSION

The functional microRNA, miR-200c, plays a significant role in many human cancers. Previous research reports that miR-200c is markedly downregulated in many cancer cells and tissues and inhibits cancer proliferation and migration [34, 35]. However, few researchers have focused on the expression of miR-200c and its biological function in breast cancer. In this study, through measuring the expression of miR-200c in breast cancer cell lines, human breast cancer tissues and their matched normal adjacent tissues, we found that miR-200c was downregulated in breast cancer. We discovered potential clinicopathological implications of miR-200c by analyzing the correlation of miR-200c levels with clinical stage, local relapse and distant metastasis. This is the first report about the clinicopathological implications arising from altered miR-200c levels in breast cancer.

We then predicted that KRAS was a target of miR-200c, which is a member of miR-200 family and usually known to function as tumor suppressors in many cancers [8, 9, 29]. miRNAs regulate gene expression by binding to the 3′-UTR of target mRNAs and either inhibiting translation or inducing degradation of mRNAs [6]. That's to say, miRNAs exert the regulation function via mRNAs or protein levels. In this study, we first detect that miR-200c levels have an inverse correlation with both KRAS protein levels and KRAS mRNA levels.

Furthermore, by over-expressing miR-200c in MDA-MB-231 and 4T1 cells, we validated that miR-200c can directly inhibit the AKT and ERK pathways by directly targeting KRAS and inhibiting its translation. The miR-200c-mimic induced downregulation of KRAS, along with decreased levels of phosphorylated AKT and phosphorylated ERK, were rescued with the introduction of KRAS. At last, we provide evidence that miR-200c can inhibit the proliferation and clone formation of breast cancer *in vitro* by targeting KRAS. These results delineated a novel regulatory network employing miR-200c and KRAS to fine-tune cell proliferation and clone formation. Further, the mechanism in which miR-200c exerts its tumor suppressive function is by targeting KRAS. Therefore, the modulation of KRAS by miR-200c may explain why the downregulation of miR-200c during breast carcinogenesis can promote cancer progression.

In summary, low miR-200c expression levels are associated with poor DFS and OS in breast cancer patients. miR-200c inhibits the AKT and ERK pathways by directly targeting KRAS. We also provide evidence about the biological effects of overexpression of miR-200c in breast cancer. We further demonstrate that miR-200c functions as a tumor suppressor in breast cancer by *in vitro* and *in vivo* models. Therefore, miR-200c is a valuable marker for breast cancer progression and prognosis.

## MATERIALS AND METHODS

### Human tissue

All the tissue samples in this study were collected from the Sun Yat-Sen University Cancer Center (Guangzhou, Guangdong, China) and used according to the ethical standards as formulated in the Helsinki Declaration. All the aspects of this study were approved by the Ethics Committee of Sun Yat-Sen University Cancer Center Health Authority.

Tissue samples from 41 breast cancer patients were subjected to qRT-PCR analysis. TMAs composed of 134 cases of breast cancer were used for *in situ* hybridization analysis, and all of them were confirmed by histopathology diagnosis during the period from October 2001 to September 2006. Resected breast cancer tissues (BC) and paired matched normal mammary tissues (CTR) were immediately cut and stored in RNAlater (Ambion). Specimens were obtained during surgery and stored in the Department of Specimens and Resources of Sun Yat-Sen University Cancer Center which were formalin fixed and embedded in paraffin following the standard methods.

All the clinical data of patients such as age, histologic type, tumor size, lymph node status, local or distant metastatic relapse, ER status, PR status and HER2 status were available and reviewed. Histologic type was determined on TNM staging system which was classified according to the WHO classification and tumor stage (American Joint Committee on Cancer classification). No patient had ever received any chemotherapy or radiation therapy before the surgery or in the present study. Follow-up included the review of records and telephone calls, and the length of follow-up was 70 months. The dates of death and relapse were used to calculate DFS and OS. DFS was calculated from diagnosis to disease progression or death, no matter which occurred first. Patients who were alive and disease free were censored at the date of last follow-up visit. Distant met referred to the cancer which spread to the whole tissues or organs, mainly including bone, lung and brain. OS was the time from diagnosis to the date of death for any cause, and patients who were alive were censored at date of last follow-up visit. The patients were grouped according to their clinical features listed in Table 1.

### *In situ* hybridization (ISH) analysis

*In situ* hybridization procedures were carried out as previously described [36]. miR-200c miRCURYTM LNA custom detection probes (Exiqon, Vedbaek, Denmark) were used for ISH. The 5′-3′ sequences (enhanced with LNA) were TCCATCATTACCCGGCAGTATTA with a DIG label at both the 5′ and 3′ ends. Hybridization, washing, and scanning were carried out according to the manufacturer's instructions. The intensities of miR-200c staining was scored by 0–4, according to the standards of 0–1 (no staining), 1–2 (weak staining), 2–3 (medium staining) and 3–4 (strong staining). The percentages of miR-200c cells in three representative high-power fields of individual samples were analyzed. Those expression scores equaled to scores of the intensities × the percentages of positive cells, and the maximum was 4 and the minimum was 0. Every samples were assessed by at least two pathologists in a blinded manner, and those expression scores of greater or equal to 2 was defined as high expression, less than 2 was low expression [37].

### Cell culture

The human breast cancer cell line MBA-MD-231 and mouse breast cancer cell line 4T1 were purchased from the Shanghai Institute of Cell Biology, Chinese Academy of Sciences (Shanghai, China). MBA-MD-231 and 4T1 cells were cultured in DMEM (Gibco, USA) supplemented with 10% fetal bovine serum (Gibco) in a humidified incubator at 37°C with 5% CO_2_.

### Quantitative real-time PCR analysis (qRT-PCR)

Total RNA from tissues and the cultured cells was extracted with the TRIzol reagent (Invitrogen, USA). Reverse transcription and qRT-PCR reactions were performed using a qSYBR-Green-containing PCR kit (Qiagen, USA) and a BioRad IQTM5 Multicolor Real-Time PCR Detection System (USA). The equation 2^−ΔΔCt^ was used to calculate the relative amount of miRNA among Which U6 snRNA was used as an internal control (Ct was the fractional cycle number at which the fluorescence of each sample passes the fixed threshold) and ΔΔCT = (C_T miRNA_ − C_T U6_)_target_ − (C_T miRNA_ − C_T U6_)_control_. The primers of KRAS mRNA for qRT-PCR detection were as follows: forward, 5′-GGACTGGG-GAGGGCTTTCT-3′ and reverse, 5′-GCCTGTTTTGTGTCTACTGTTCT-3′. The primer was synthesized by Invitrogen. Each experiment was performed three times intriplicate.

### miRNA overexpression

miRNA mimic (a synthetic RNA oligonucleotide duplex mimicking miRNA precursor) was used for the overexpression of miRNA. The synthetic RNA molecules were purchased from GenePharma (Shanghai, China) including pre-miR-200c and pre-miR-control (scrambled negative control RNA). MDA-MB-231 and 4T1 cells seeded in 6-well plates were transfected with Lipofectamine 2000 (Invitrogen) when the cells were nearly 70% confluent. Then 100 pmol of pre-miR-200c was added. 6 h later, changed the culture medium to DMEM supplemented with 2% fetal bovine serum (FBS). 24 h or 48 h after the transfection, harvested the cells for the isolation of total RNA or protein.

### Recombinant vector

Recombinant lentiviruses containing miR-200c precursor or scramble sequences were purchased from SunBio (Shanghai, China). pReceiver-M02-KRAS (a mammalian expression plasmid) designed to specially express the human KRAS’ full-length open reading frame (ORF) without the miR-200c-responsive 3′-UTR was purchased from GeneCopoeia (Germantown, USA). An empty plasmid was served as a negative control. Overexpression plasmid was transfected into MDA-MB-231 and 4T1 cells by Lipofectamine 2000 (Invitrogen) following the manufacturer's instructions. 24 h or 48 h after transfection, total RNA or protein was isolated respectively. The expression levels of KRAS mRNA and protein were evaluated by qRT-PCR and Western blotting.

### Luciferase reporter assay

The full-length of 3′-UTRs of the KRAS gene was amplified by PCR from MDA-MB-231 genomic DNA and inserted into pGL3 control vector (Promega, WI). Using the QIAGEN XL-site directed Mutagenesis Kit (QIAGEN, CA), we generated several inserts by deletions of 4 bp from the perfectly complementarity site of KRAS gene. According to the manufacturer's instructions (solution V, programme T-016), MDA-MB-231 cells were cotransfected with 0.5 ug firefly luciferase report vector and 0.5 ug control vector containing Renilla luciferase, pRL-TK (Promega) by nucleoporation(AmaxaBiosystems). Each nucleoporation used 50 nM of the miR-200c or a scrambled oligonucleotide. 48 hours after transfection, the relative luciferase activities (RLA) of Firefly and Renilla were consecutively measured through the dual luciferase assay (Promega).

### Protein isolation and western blotting

Protein was extracted fromMDA-MB-231 and 4T1 cells lines by RIPA lysis buffer with proteinase inhibitor. By Protein BCA Assay Kit (Bio-Rad, USA), the concentration of protein in the lysates was measured. Each lane was loaded with 20 μg protein mixed with 2 × SDS loading buffer. The protein was separated by 12% SDS-polyacrylamide gel electrophoresis, and then transferred to polyvinylidenedifluoride membranes (Millipore, USA). The membranes were incubated with 5% skim milk powder at room temperature for 1 h to block nonspecific binding. Subsequently, the membranes were incubated with antiserum containing antibodies against KRAS (F234), AKT(sc-1619), p-AKT (sc-33437), ERK (sc-292838) and p-ERK (sc-32577) which were purchased from Santa Cruz Biotechnology (Santa Cruz, CA) at 4°C for 12 h. To visualize the target proteins, a 1:5000 dilution of peroxidase-conjugated secondary antibody and ECL Western blotting detection reagents (ECL New England Biolabs, USA) were used. A Bio Image Intelligent Quantifier 1-D was used to quantify (Version 2.2.1, Nihon-BioImage Ltd., Japan). Also an anti-β-actin antibody as a protein loading control (Boster, Wuhan, China) was used.

### Cell proliferation assay

Cell viability analysis was preformed according to the methods using 3-(4, 5-Dimethylthiazol)-2, 5-Diphenyltetrazolium Bromide [38]. To assess the incorporation of BrdUrd, MDA-MB-231 and 4T1 cells in treated and control term were incubated with 10 nM BrdUrd contained culture media for 16 h, then the cells were fixed with cold methanol and acetone (1:1).

### Survival foci formation assay

Cells in exponential growth phase were plated into a six-well plate at 2000 cells/well and treated with miRNA after adhesion. When most cell clones had reached >50 cells, they were stained with 0.06% crystal violet, and foci number was counted.

### Mouse xenograft model

As previously described [39], the breast cancer model in BALC/c mice was established. 2 × 10^5^ 4T1 cells infected with miR-200c precursoror scramble were inoculated subcutaneously into the dorsal flanks of mice (five in each group). Every four days the tumor size was measured. 28 days later, we killed the mice and performed necropsies. Then the tumors were weighed. The tumor volumes were calculated following the formula: A × B^2^/2 (A: the largest diameter, B: the diameter perpendicular to A). All the procedures involved were performed following institutional guidelines.

### Statistical analysis

All the images of Western blotting assay were the representative of at least three times independent experiments. qRT-PCR, the luciferase reporter, survival foci formation and cell proliferation assay were performed in triplicate. Each experiment was repeated for several times and the results were presented as the means ± SE. Statistical analysis was analyzed with a Student's *t*-test. According to the Kaplan-Meier method, Overall survival (OS) and disease free survival (DFS) curves were plotted. The significance level was set as *p* < 0.05 level. All the statistical analysis was accomplished by the SPSS 17.0 software.

## SUPPLEMENTARY FIGURES



## Abbreviations

ER: estrogen receptor
PR: progesterone receptor
HER2: human epidermal growth factor receptor-2
EGFR: epidermal growth factor receptor
