# Supplementary material for: miR-200c inhibits breast cancer proliferation by targeting KRAS
Source: Oncotarget. 2015 Sep 9;6(33):34968–78. doi: 10.18632/oncotarget.5198 (PMC4741502; doi:10.18632/oncotarget.5198)
Supplement: Supplementary file 1 [file oncotarget-06-34968-s001.pdf]

SUPPLEMENTARY FIGURES

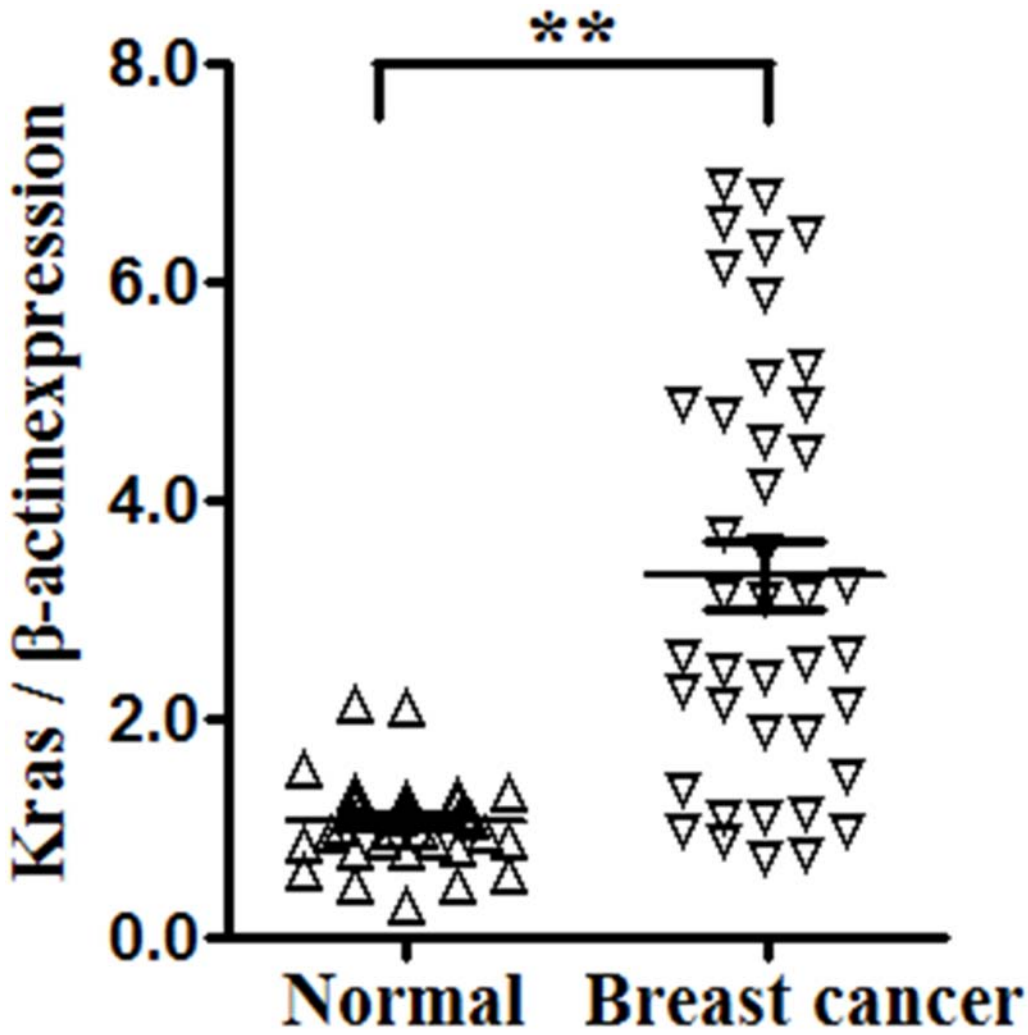

Supplementary Figure S1: Expression levels of KRAS in 41 pairs of breast cancer tissues (BC) and their matched normal adjacent tissues (CTR). The relative expression levels of KRAS were determined by quantitative RT-PCR. All of the data are shown as the means ± s.e.m. $^{**}P < 0.01$.

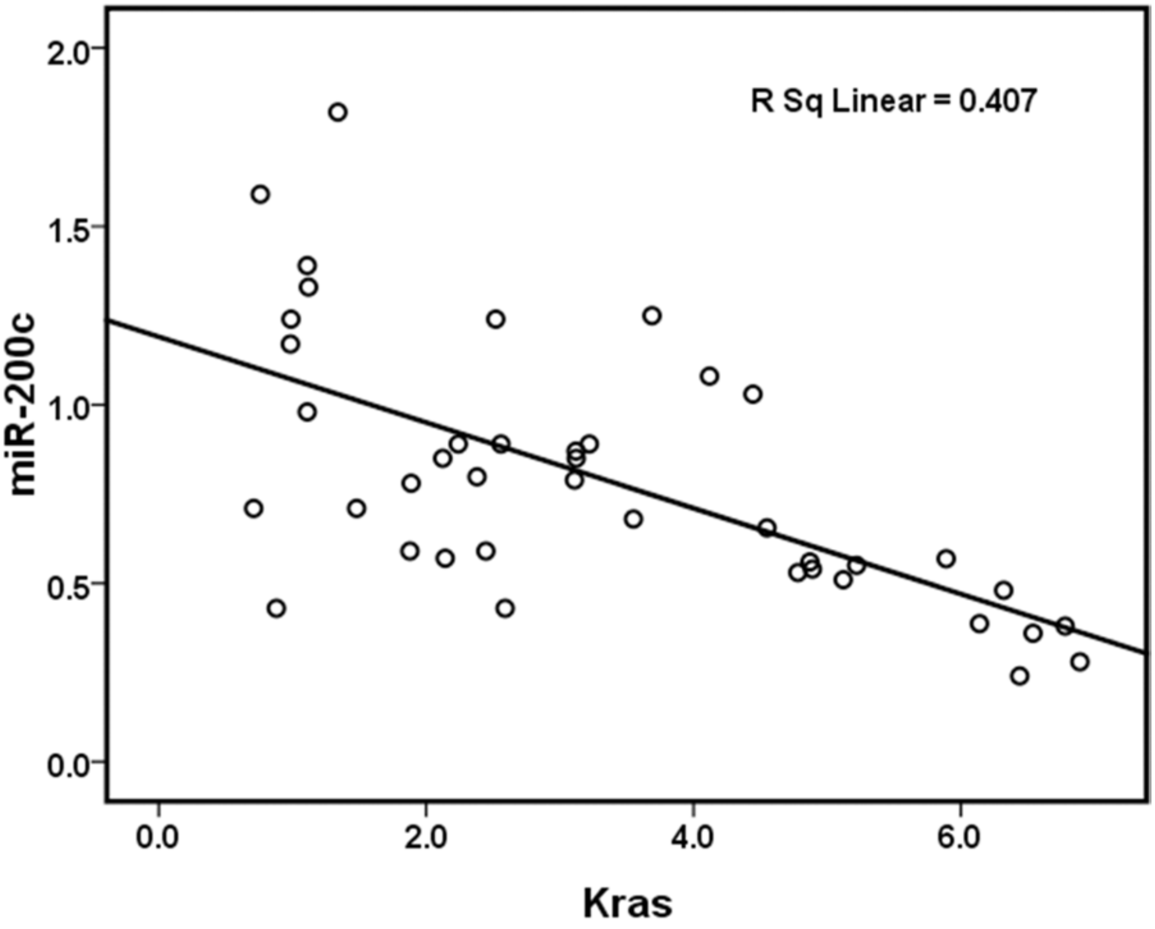

Supplementary Figure S2: Statistical analysis of the correlation between the expression of miR-200c and KRAS. miR-200c levels in 41 breast cancer tumors samples were significantly inversely correlated with the expression of KRAS. All of the data are shown as the means ± s.e.m. $^{**}P < 0.01$.

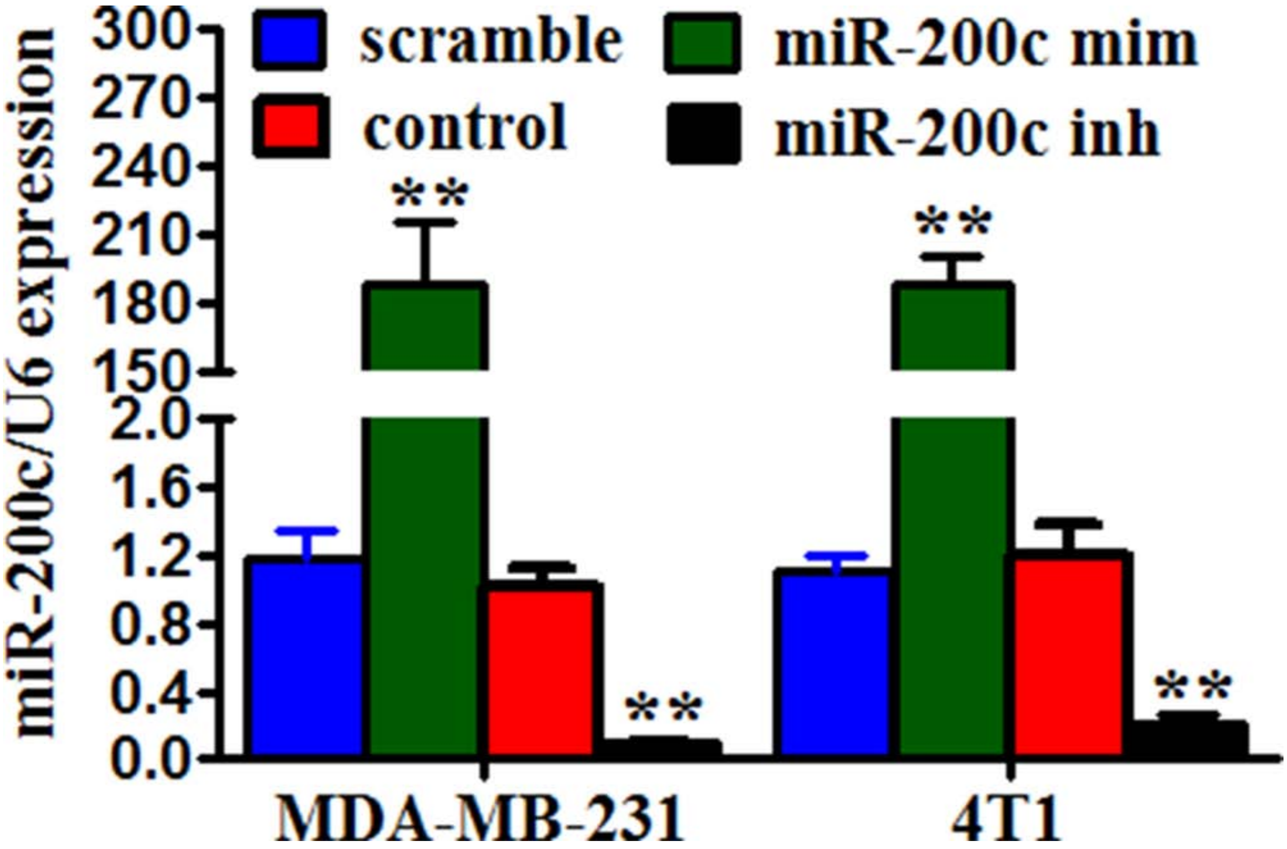

Supplementary Figure S3: The transfection was successful. MDA-MB-231 and 4T1 cells were transfected with miR-200c mimics, miR-200c inhibitors or their scrambled oligonucleotides controls. All of the data are shown as the means ± s.e.m. $^{**}P < 0.01$.

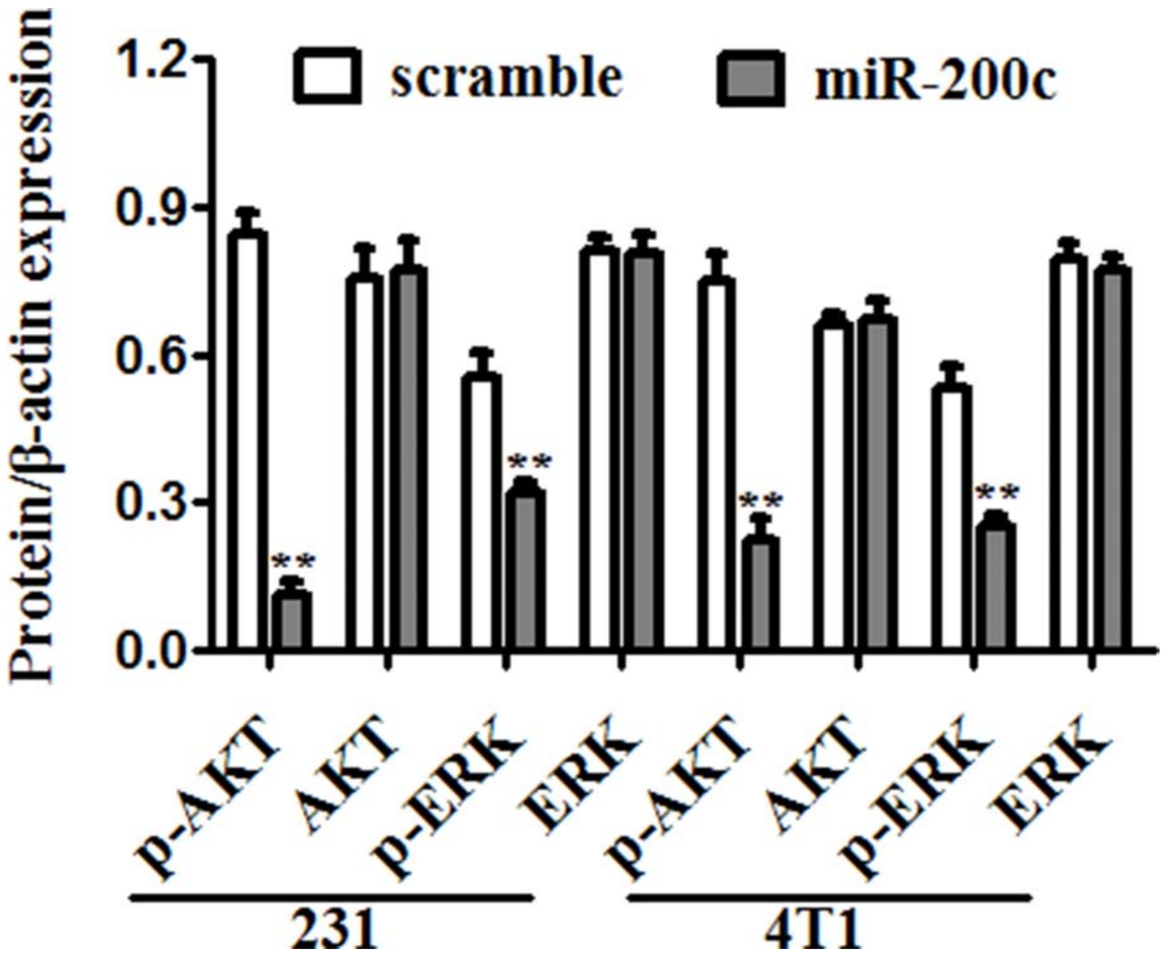

Supplementary Figure S4: Quantification of protein levels in Figure 3E. Quantification of AKT, ERK, pAKT and pERK protein levels in MDA-MB-231 and 4T1 cells was analysed by Image J. All of the data are shown as the means ± s.e.m. $**P < 0.01$.

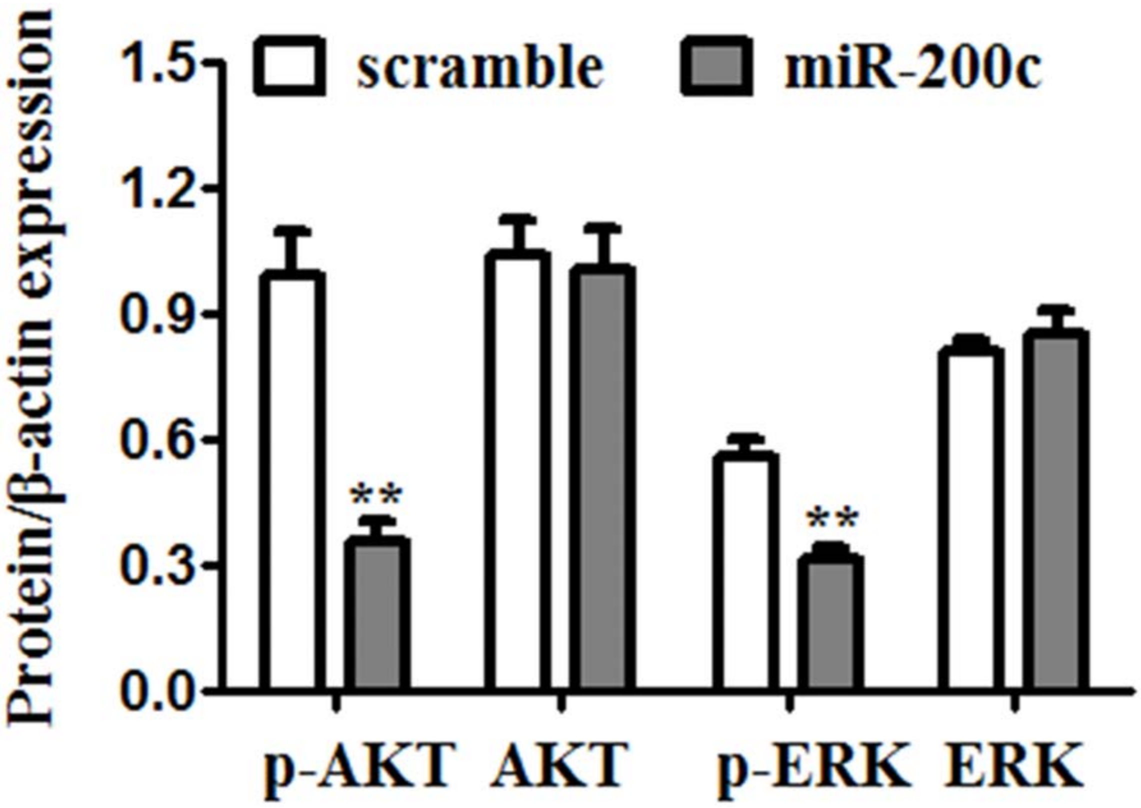

Supplementary Figure S5: Quantification of protein levels in Figure 3G. Quantification of AKT, ERK, pAKT and pERK protein levels in 4T1 cells was analysed by Image J. All of the data are shown as the means ± s.e.m. $^{**}P < 0.01$.
